# Protective effect of natural products in the metabolic-associated kidney diseases *via* regulating mitochondrial dysfunction

**DOI:** 10.3389/fphar.2022.1093397

**Published:** 2023-01-12

**Authors:** Peng Liu, Yao Chen, Jing Xiao, Wenhui Zhu, Xiaoming Yan, Ming Chen

**Affiliations:** ^1^ Shunyi Hospital, Beijing Traditional Chinese Medicine Hospital, Beijing, China; ^2^ Department of Medicine, Renal Division, Heilongjiang Academy of Chinese Medicine Sciences, Harbin, China; ^3^ Department of Medicine, Digestive Division, Heilongjiang Academy of Chinese Medicine Sciences, Harbin, China

**Keywords:** metabolic-associated kidney diseases, mitochondrial dysfunction (MD), natural products, diabetic nephropathy, obesity-related nephropathy, hypertensive kidney disease, gouty nephropathy

## Abstract

Metabolic syndrome (MS) is a complex group of metabolic disorders syndrome with hypertension, hyperuricemia and disorders of glucose or lipid metabolism. As an important organ involved in metabolism, the kidney is inevitably attacked by various metabolic disorders, leading to abnormalities in kidney structure and function. Recently, an increasing number of studies have shown that mitochondrial dysfunction is actively involved in the development of metabolic-associated kidney diseases. Mitochondrial dysfunction can be used as a potential therapeutic strategy for the treatment of metabolic-associated kidney diseases. Many natural products have been widely used to improve the treatment of metabolic-associated kidney diseases by inhibiting mitochondrial dysfunction. In this paper, by searching several authoritative databases such as PubMed, Web of Science, Wiley Online Library, and Springer Link. We summarize the Natural Products Protect Against Metabolic-Associated Kidney Diseases by Regulating Mitochondrial Dysfunction. In this review, we sought to provide an overview of the mechanisms by which mitochondrial dysfunction impaired metabolic-associated kidney diseases, with particular attention to the role of mitochondrial dysfunction in diabetic nephropathy, gouty nephropathy, hypertensive kidney disease, and obesity-related nephropathy, and then the protective role of natural products in the kidney through inhibition of mitochondrial disorders, thus providing a systematic understanding of the targets of mitochondrial dysfunction in metabolic-associated kidney diseases, and finally a review of promising therapeutic targets and herbal candidates for metabolic-associated kidney diseases through inhibition of mitochondrial dysfunction.

## Introduction

Metabolic-Associated Kidney Diseases are a variety of kidney diseases caused by metabolic disorders, such as diabetic kidney disease (DKD), gouty nephropathy (GN), hypertensive kidney disease and obesity-related glomerular disease (ORG). High hemodynamic, excretory overload, endothelial dysfunction, chronic inflammation, and other mechanisms (Dugan et al., 2013) act alone or in interaction to cause kidney injury (Inagi, 2022) The prevention and treatment of such kidney diseases requires a multifactorial approach. Weight loss *via* diet control and exercise can reverse many pathophysiologic processes (Mafra et al., 2018). Pharmacological interventions include strict glycemic and lipid control (Felizardo et al., 2014), blockade of the renin-angiotensin-aldosterone system (RAAS) (de Cavanagh et al., 2011) and anti-inflammatory treatments (Bhatia et al., 2020).

Diabetic nephropathy is defined as a chronic kidney disease (CKD) caused by diabetes, The basic pathological alterations include interstitial inflammation and fibrosis, thickening of the glomerular basement membrane, mesangial matrix hyperplasia, fusion or loss of podocytes, and tubular atrophy. Functionally, the main manifestations are hyperfiltration, hypoperfusion, and alteration of the glomerular filtration barrier. However, it is known to be influenced by genetic susceptibility factors, renal hemodynamics, metabolic issues, inflammatory responses, oxidative stress, endoplasmic reticulum stress, autophagy. However, as the pathogenesis of DKD continues to be explored, studies are finding a subtle relationship with mitochondria in the kidney perhaps early in the course of diabetic nephropathy or even during diabetes (Higgins and Coughlan, 2014).

The kidney is highly aerobic and rich in mitochondria. Evidence suggests that mitochondrial dysfunction plays a crucial role in the pathogenesis of various renal diseases, especially diabetic nephropathy (Liu et al., 2017). Mitochondria are highly dynamic and morphologically plastic organelles in cells with their own unique DNA, consisting of four parts: inner membrane (IMM), outer membrane (OMM), membrane gap (IMS), and matrix, with functions such as cellular respiration, production of reactive oxygen species (ROS) and oxidative phosphorylation (OXPHOS) to produce adenosine triphosphate (ATP) (Mills et al., 2022). Once mitochondria are damaged, not only are there morphological and functional changes, but also the accumulation of damaged mitochondria can result in several pathological changes, including the accumulation of by-products ROS, a decrease in mitochondrial membrane potential, and the translocation of apoptotic proteins (Westermann, 2010). When mitochondria are damaged, they not only undergo morphological and functional changes, but various symptoms can manifest at various locations. In the case of GN (Hong et al., 2012), hypertensive kidney disease (Eirin et al., 2015), and ORG (Nemmar et al., 2017), also caused by metabolic factors, mitochondrial dysfunction is involved and has a great impact on the pathology and physiology (Yan et al., 2020).

In this review, we will summarize natural products in the treatment of metabolic-associated kidney diseases, beginning with the pathogenesis of mitochondrial dysfunction.

## Protective effect of natural products in diabetic nephropathy *via* regulating mitochondrial dysfunction

### Protective effect of natural products in diabetic nephropathy *via* improving respiratory chain abnormalities

Mitochondria are the main site of aerobic cellular respiration, and this is accomplished mainly by the mitochondrial respiratory chain. Mitochondrial respiratory chain, also known as the electron transport chain (ETC), consists of four complexes I, II, III, and IV. There are two known ETCs, one is the nicotinamide adenine dinucleotide (NADH) respiratory chain, NADH-complex I-coenzyme Q-complex III-cytochrome c (Cyto c)-complex IV-O2, and the other is the reduced flavin adenine dinucleotide (FADH2) respiratory chain, FADH2-complex II-coenzyme Q-complex III- Cyto c complex IV-O2. On this basis, the mitochondrial inner membrane respiratory chain enzyme system is further divided into five major groups: complexes I, II, III, IV, and complex V (ATPase) which is not part of the respiratory chain (Ahmad et al., 2021).

Resveratrol (RESV) (Zhang B. et al., 2019) and Curcumin (CUR) can increase Complex I and III activity and improve the respiratory chain and thus mitochondrial dysfunction. CUR also elevates the TCA cycle (Han et al., 2021) and restores the mitochondrial coupling process and the abnormal opening of the mitochondrial permeability transition pore.

As the end product of glycolysis, pyruvate is located at the intersection of glycolysis and the tricarboxylic acid cycle, and the Mitochondrial pyruvate carrier (MPC) located in the inner mitochondrial membrane transports pyruvate from the cytoplasm to the mitochondrial matrix (Taylor, 2017), abnormalities in MPC will affect the uptake and utilization of pyruvate by the mitochondria, thus affecting the normal function of the mitochondrial respiratory chain. The expression of peroxisome proliferator-activated receptor-g coactivator 1a (PGC-1α), MPC1, and MPC2 was increased by Artemether in db/db mice (Han et al., 2019). PDK1 is a kinase that inactivates pyruvate dehydrogenase (Klyuyeva et al., 2019), and Artemether treatment was found to reduce the level of mitochondrial protein PDK1, reverse the low expression of catalase, SOD2, and other antioxidant enzymes, and alleviate the redox imbalance in type 1 diabetes (T1D) DKD mice (Wang M. et al., 2019).

The mitochondrial complex III rapidly produces ROS in the sensitive area of antimycin A, and melatonin can act as a ROS scavenger. Studies have shown (Zhang et al., 2011) that melatonin can increase the expression of the complex I protein in db/db mice, reduce complex III activity, and damage mitochondria by affecting the balance of mitochondrial electron transport, leading to overproduction of ROS. Melatonin restores mitochondrial membrane potential in podocytes, dose-dependent elimination of Angiotensin II-induced ROS increase, and protects damaged mitochondria (Ji and Xu, 2016). In addition, Melatonin reduced the abnormal increase in ROS and inhibited the high expression of pro-apoptotic proteins [caspase-3, -9 and Bcl-2-associated X protein (Bax)], possibly due to a protective mechanism mediated by the 5-AMP-activated protein kinase (AMPK)/PGC-1α pathway (Satari et al., 2021).

The activity of antioxidant enzymes is sometimes also closely related to mitochondrial respiratory chain abnormalities. Purslane polysaccharide (Wang and Ye, 2018) and Jujuboside A (Zhong et al., 2022) both increase the activity of mitochondrial respiratory chain complexes and antioxidant enzymes, restoring damage caused by mitochondrial respiratory chain abnormalities as well as oxygen species (OS).

### Protective effect of natural products in diabetic nephropathy *via* inhibiting oxidative stress and inflammatory response

Abnormalities in the mitochondrial respiratory chain can lead to an abnormal increase in one of the final harmful products, ROS. The products of ROS mainly include to the single electron reduction product of O2, superoxide anion (O2-), and its derivatives hydrogen peroxide (H2O2), hydroxyl radical (OH-), and singlet state O2 (Ahmad et al., 2021). An increase in ROS production and/or a decrease in the ability to clear ROS for various reasons can lead to an increase in endogenous ROS, which can leads to a state of oxidative and antioxidant imbalance, that is, what we often call OS. Moreover, increased ROS leads to breakage of mtDNA and interferes with transcriptional synthesis, which affects the efficacy of ETC and leads to reduced ATP production; it also stimulates inflammatory factors and thus induces an inflammatory cascade response; increased ROS triggers autophagy in the mitochondrial pathway, activates apoptosis-related proteins, and triggers apoptosis in the mitochondrial pathway (Ruiz et al., 2013; Flemming et al., 2016).

Antioxidant enzymes can counteract the abnormal increase of ROS. Mn-SOD is one of the few antioxidant enzymes present in mammalian mitochondria. A study (Williams et al., 1998) provides direct evidence that reduced Mn-SOD activity *in vivo* is associated with increased mitochondrial oxidative damage and consequently altered function. As ROS scavengers, RESV can increase the activity of Mn-SOD and other mitochondria-associated peroxidases and thus reduce the damage caused by OS to mitochondria, and in addition, the expression of 8-OHdG in mtDNA and the accumulation of D-17 deletion in mtDNA are correspondingly reduced after RESV treatment (Kitada et al., 2011).

RESV Zhang T. et al. (2019), Berberine (BBR) (Qin et al., 2019), cyanidin-3-O-β-glucoside chloride (C3G) (Wei et al., 2018) can eliminate mitochondrial ROS and reverse the abnormal changes in mitochondria caused by OS. Inhibition of the NRF2/HO-1 pathway can alleviate OS, and both Notoginsenoside R1 (Zhang B. et al., 2019) and Obacunone (OB) (Zhou et al., 2019) can eliminate ROS through this pathway and attenuate the damage caused by OS. Astaxanthin (ASX) scavenges excess ROS as well as ROS-modified protein 4-hydroxy-2-nonenal (4HNE) from mitochondria in Normal human mesangial cells (NHMCs), and ASX inhibits the development of diabetic nephropathy by reducing mitochondrial damage through Inhibition of ROS production (Manabe et al., 2008). Curcumin inhibits protein kinase C-β(PKCβ)/p66Shc/NADPH oxidase and activates FOXO-3a to suppress OS in the mitochondrial pathway through a decrease in p66Shc levels in mitochondria (Altamimi et al., 2021).

Silent information regulator 1 (SIRT1) is a histone deacetylase dependent on nicotinamide adenosine dinucleotide (NAD^+^). Puerarin (PR) ameliorates varying degrees of mitochondrial damage under the ultrastructure of DN mice kidney tissue cells. Activation of the SIRT1/(forkhead transcription factor-O1) FOXO1 pathway would result in low OS in mitochondria. PR positively upregulates SIRT1/FOXO1 signaling pathway expression levels, significantly reducing the expression of inflammatory factors such as NF-κB, IL-6, and TNF-α. PR exerts antioxidant effects by increasing the activity of Mn-SOD and CAT. Above all, it improves mitochondrial dysfunction by alleviating OS and inflammatory response (Xu et al., 2016). It was found that the accumulation of ceramide could lead to elevated mitochondrial ROS. Myriocin, as an inhibitor of *de novo* ceramide synthesis, could reduce ceramide and subsequent ROS accumulation, and myriocin treatment could reduce vesicle expansion of mitochondrial cristae and maintain the stability of mitochondrial morphology, as observed under Electron Microscope (Woo et al., 2020).

OS and inflammation are closely linked and both contribute to the progression of DKD (Bruchfeld et al., 2016). To some extent, the inflammatory response is a kind of self-protection of the body, however, the long-term persistent inflammatory state is a harm to the body. The most studied and inextricably linked to mitochondrial dysfunction is the NOD-like receptor protein 3 (NLRP3) inflammatory vesicles, which are multiprotein complexes. There are multiple pathways for NLRP3 inflammatory vesicle activation, one of which is: being oxidized mtDNA fragments enter the cytoplasm *via* the mitochondrial permeability transition pore (mPTP) and then bind to NLRP3 and cyclic GMP-AMP Synthase (cGAS) to activate NLRP3 inflammasomes. A study has confirmed the key role of ROS in activating small inflammatory bodies in diabetic kidney disease (Shahzad et al., 2015). CUR Lu et al. (2018) and Icariin (Ding et al., 2022) both inhibit the activation of NLRP3 inflammasome.

The activation of NLRP3 further activates downstream inflammatory factors to aggravate kidney injury, and ROS, as one of the common activation pathways of NLRP3, his activation mechanism is gradually coming into the public view, but the current research reports on this area are few and not in-depth, and there are many anti-inflammatory herbs and active ingredients in Chinese traditional medicine, which we believe will bring us more surprises in the future.

### Protective effect of natural products in diabetic nephropathy *via* regulating mitochondrial quality control

Mitochondria are in a dynamic and highly mobile state, and mitochondrial quality control (MQC) is an effective way for mitochondria to maintain their dynamic homeostasis, which mainly includes mitochondrial dynamics, mitochondrial biogenesis, mitochondrial autophagy and other processes. Mitochondrial dynamics includes mitochondrial fusion and fission which redistributes mitochondrial components, prevents defective mitochondria from continuing to fuse and helps restore damaged mitochondrial membrane activity. Optic atrophy1 (OPA1) (Westermann, 2010; Luo et al., 2015). Astragaloside II (AS-II) (Su et al., 2021) has been found to improve mitochondrial dynamics by upregulating Mitofusin 2 (MFN2) expression and decreasing Fission 1 (FIS1) expression in DKD rats studied *in vivo*. MFN2 is a protein involved in mitochondrial membrane fusion. FIS1 is located in the mitochondrial membrane and regulates mitochondrial division.

Mitochondrial fission can separate damaged mitochondria prior to mitochondrial autophagy (Yang et al., 2017). Its related proteins include mitochondrial dynamin-related proteins (DRPs) and mitochondrial fission family, *etc.* DRP1 is a large GTPase, that is, translocated from the cytoplasmic matrix to the OMM, forming a ring-like oligomeric structure around the fragmented membrane. After mitochondrial fission, DRP1 returns from the OMM to the cytoplasmic matrix. Decreasing DRP1 expression enhances mitochondrial fusion and forms enlarged mitochondria, while increasing DRP1 expression results in mitochondrial fragmentation. FIS1 phosphorylated on OMM binds to DRP1 and co-regulates mitochondrial fission. In addition, FIS1 can eventually trigger apoptosis through the induction of Cyto c (Lee et al., 2004). A study Liu et al. (2017) showed that Astragaloside IV (AS-IV) significantly improved the down-regulated expression of DRP-1, FIS-1 in a db/db mouse model. *In vitro* and *in vivo* experiments have demonstrated that BBR can phosphorylate DRP1 and use mitochondrial fission to eliminate excess mtROS (Kim and Lee, 2021). BBR may improve mitochondrial dynamics by blocking Drp1 expression and translocation to mitochondria in lentivirus-infected foot cells expressing DRP1 (Qin et al., 2019). PD can improve mitochondrial function and morphological changes through ROS/DRP1/mitochondrial fission, and this pathway is expected to be a new therapeutic target (Ni et al., 2017). Melatonin improves kidney mitochondrial dynamics by inhibiting fission and promoting fusion, as well as improving mitochondrial physiology, thus enhancing kidney function in diabetic obese rats. Promote fusion protein MFN2 and OPA1 expression, promote fusion, and restore fission protein DRP1 to normal. Increase compound IV activity (Agil et al., 2020).

Mitochondrial Pathway Autophagy is a selective autophagy process in which damaged and senescent mitochondria are specifically identified and cleared under stressful conditions to maintain internal environmental stability (Green and Van Houten, 2011; Novak, 2012). PTEN induced putative kinase 1 (PINK1) is a parkin-recruitment factor from the cytoplasmic to damage mitochondria in a membrane potential-dependent manner for mitochondrial degradation (Matsuda et al., 2010). PINK/Parkin/P62 is a classical pathway for mitochondrial autophagy, and PINK1 is located upstream of Parkin. When the membrane potential decreases, Parkin is activated and displaced, cytosolic p62 translocates to the mitochondria and binds to the polymerized ubiquitin-binning domain, which then binds to LC3-interacting region (LIR) to facilitate autophagy completion (Gottlieb et al., 2021). The study found that AS II (Liu et al., 2017) and Jujuboside A (Zhong et al., 2022) downregulated PINK1/Parkin-mediated mitochondrial autophagy to improve mitochondrial dysfunction. Sestrin2 (Sesn2) is a highly conserved protein that can be derived under a variable of stress conditions, which has been shown to regulate AMPK activation to maintain mitochondrial functional integrity and reduce ROS production. *In vitro* and *in vivo* studies demonstrated that Icariin (ICA) was associated with increased Sesn2-mediated mitochondrial autophagy and reduced NLRP3 inflammatory small-body activation in the Sesn2/PINK1/Parkin signaling pathway (Ding et al., 2022).

In addition, there also have other Parkin independent pathways and mitochondrial autophagy mediated by receptors such as NIX/BNIP3L, BNIP3, and FUNDC1. But there are no studies as extensive as the Parkin-dependent pathway described above.

Mitochondrial biogenesis is a continuous process of producing new mitochondria; PGC-1α is the primary upstream regulator of mitochondrial biogenesis, and AMPK and SIRT1 is its upstream regulator. Because PGC-1α is almost ubiquitous, targeting its upstream regulatory receptors is often considered an important method for restoring mitochondrial function (Ruozhi et al., 2021). Grape seed proanthocyanidins extract (GSPE) restores mtDNA content and improves mitochondrial dysfunction by activating the AMPK-SIRT1-PGC-1α signaling pathway (Bao et al., 2014). RESV (Zhang T. et al., 2019) and Salidroside (Xue et al., 2019) enhance the activity of the SIRT1 and PGC-1α pathway, promote mitochondrial biosynthesis, inhibit mitochondrial OS, and improve kidney damage. PGC-1α promotes mitochondrial transcription factors such as nuclear response factor 1/2 (NRF1/2) and transcription factor a mitochondrial (TFAM). RESV (Kitada et al., 2011) and BBR (Qin et al., 2019) reversed the low expression of PGC-1α and the downstream factors described above, increasing mtDNA content and improving mitochondrial biogenesis. Syringic Acid (SYR) (Rashedinia et al., 2020; Rashedinia et al., 2021) reverses low mRNA expression of PGC-1α and NRF-1 in diabetic rats, increases mtDNA/nDNA ratio, decreases antioxidant enzyme activity such as CAT, and regulates mitochondrial biosynthesis and oxidative stress to mitigate mitochondrial damage. RESV may enhance mitochondrial biogenesis by reducing OS (Kitada et al., 2011). SIRT3, a member of the SIRT family, is primarily present in the linear granule matrix and also regulates the indirect mitochondrial functions of PGC-1α, Complex I, II, and SOD2 (Morigi et al., 2018). Honokiol restored Sirt3 expression in BTBR ob/ob mice, inhibited OS through SIRT3, SOD2, restored PGC-1α levels, and improved mitochondrial morphology and function (Locatelli et al., 2020).

### Protective effect of natural products in diabetic nephropathy *via* substance alteration

Mitochondria are the only organelles with unique genomes (mtDNA) (Gottlieb et al., 2021). Mitochondria include nuclear DNA (nDNA) and mtDNA. Compared to nDNA, mtDNA lacks an effective DNA damage repair system and histone protection, making mtDNA more susceptible and difficult to repair (Luo et al., 2015). MtDNA is exposed to respiratory chains and mitochondrial membranes, making it more sensitive to OS, and mtDNA and oxidative stress interact: abnormal increases in ROS lead to mtDNA breaks, which in turn induces an inflammatory response that increases ROS and further exacerbates damage if mtDNA oxidizes. Because abnormal mitochondrial function continues to affect mtDNA integrity; Therefore, we have to consider how much mtDNA is involved in almost any manifestation of mitochondrial abnormalities. The previously mentioned monomer components of RESV, SYR, GSPE, and Salidroside all reverse damage caused by mtDNA acting in concert with other mechanisms of mitochondrial dysfunction.

When ETC synthesizes ATP, a large number of protons are produced, and the mitochondrial membrane potential (MMP) caused by this concentration difference also provides the power for mitochondrial uptake of cytosolic Ca^2+^, a selective pathway for mitochondrial matrix uptake called mitochondrial Ca^2+^ uniporter (MCU). Overexpression of MCU led to increased mitochondrial Ca^2+^ and decreased cytoplasmic Ca^2+^, leading to decreased MMP and increased abnormal openness of mPTP. In fact, at least three dehydrogenases in the TCA cycle are regulated by the mitochondrial substrate Ca^2+^, which mediates oxidative phosphorylation. Due to Ca2^+^-ATP enzyme regulation, moderate calcium in the mitochondria promotes ATP synthesis, while calcium overload leads to mPTP opening, decreased membrane potential, and apoptotic protein spillovers, so regulating calcium balance plays an important role in MQC. Ca^2+^ throughput chemiluminescence (CHL) assay confirmed increased oxygen consumption and ROS production in isolated mitochondria (Kanno et al., 2004).

AS-IV dose-dependent increase in Sarco/endoplasmic reticulum Ca^2+^-ATPase (SERCA) activity, particularly its subtype SERCA2, evened out intracellular Ca^2+^, Ca^2+^ induces typical mitochondrial membrane permeability transition (MPT), which results in abnormal opening of mPTP, depolarization and swelling of mitochondria, and finally release Cyto c into the cytoplasmic matrix. AS-IV presumably by activating the PPAR-γ/SERCA2/Ca^2+^ pathway, inhibiting mitochondrial pathway-mediated podocytes apoptosis as well as ER-mediated apoptosis pathway (Guo et al., 2016). Canadian Transient Receptor Potential6Channel (TRPC 6) is a member of the Ca^2+^ transduction pathway in the TRPC family and is widely expressed in kidney cells, regulating podocytes by regulating calcium homeostasis. Taurine reduces mitochondrial ROS production by increasing hydrogen sulfide synthesis, increasing respiratory chain complex expression, modulating the CSE/TRPC6 axis to inhibit calcium overload, and improving mitochondrial respiratory function, ultimately reducing DN damage (Zhang et al., 2020).

Hyperglycemic state can lead to an increase in the IMM potential difference and the formation of inner membrane hyperpolarization, resulting in mitochondrial dysfunction. RESV (Yang and Zhou, 2019; Zhang B. et al., 2019), Notoginsenoside R1 (Zhang T. et al., 2019) and Luteolin (Yu et al., 2019) concentration dependent increase mitochondrial membrane potential and decrease mitochondrial damage.

OB Zhou et al. (2019) inhibits ROS production to maintain mitochondrial membrane potential stability. Mitochondria are membrane structures with high levels of unsaturated fatty acids and are vulnerable to ROS, making mPTP abnormally open, which in turn increases membrane permeability. Small molecular mass proteins continuously enter the IMM due to the hyperosmotic state of the matrix and the ion concentration gradient on both sides of the IMM disappears, ultimately leading to poor outcomes. The breakdown of MMP can occur through uncoupling of respiratory chains, and ATP synthesis is blocked, but the mechanism of this pathway needs to be further elucidated. Higher concentrations of positive ions in the mitochondrial matrix than in the cytoplasm can exacerbate swelling and even rupture mitochondria (Zhang et al., 2021). CUR relieves impaired renal mitochondrial coupling and slows MTPTP activation in rats. C3G (Wei et al., 2018) can reverse the abnormal increase of mitochondrial membrane potential, improve OS and inhibit apoptosis. Different causes of MMP decline led to increased membrane permeability, activation of apoptosis-related proteins, and apoptosis.

### Protective effect of natural products in diabetic nephropathy *via* regulating apoptosis

Apoptosis is a genetically controlled process of programmed cell death, an active suicide attempt to maintain normal tissue morphology and function. Apoptotic cells showed typical morphological changes, including follicles, nuclear consolidation and sequenced DNA fragmentation *etc.* Mitochondrial pathway apoptosis is a type of apoptosis, mainly characterized by a decrease in MMP and an increase in mitochondrial membrane permeability. Mitochondrial pathway apoptosis includes anti-apoptotic protein B-cell lymphoma-2 (Bcl-2) family (Kale et al., 2018) and the pro-apoptotic protein Bax, which eventually activates pro-caspase9 and subsequently cascades into caspase-family cascades that eventually break down into apoptotic small bodies. Cyto c located outside the mitochondrial endometrium, not only mediates electron transfer of the respiratory chain, but also enters the cytoplasm in large quantities when cells are damaged and induces apoptosis (Bock and Tait, 2020).

Notoginsenoside R1 (Zhang B. et al., 2019), Polydatin (PD), BBR (Qin et al., 2019), OB (Zhou et al., 2019) C3G, Taurine (Das and Sil, 2012) and CUR (Altamimi et al., 2021) all reduce mitochondrial damage by regulating this mitochondrial apoptotic cascade pathway. Smac-DIABLO is a newly discovered mitochondrial apoptosis protein in these years. RESV reverses the release of Cyto c and Smac-DIABLO, activates caspase 3, and reduces damage caused by aberrant apoptosis. Mangiferin (MGF) inhibits TNFα-mediated apoptosis by reducing levels of released TNFα. MGF also reduced Cyto c release and reversed poly (ADP-ribose) polymerase (PARP) expression regulating apoptosis in the mitochondrial pathway (Pal et al., 2014). Phillyrin increased levels of Bcl-2 and Cyto c in mitochondria by regulating the PI3K/Akt/GSK-3β pathway, and decreased expression of cleaved caspase-3 and Bax proteins to regulate apoptosis in mitochondrial pathways (Wang et al., 2021). In HG-induced MPC-5 cells, Luteolin treatment significantly upregulated the expression of the mitochondrial apoptotic protein Bcl-2 and downregulated the expression of caspase proteins. Luteolin interfered in a similar manner to siNLRP3, inhibiting the formation of NLRP3 inflammatory small bodies and acting as an anti-apoptotic agent (Yu et al., 2019). Apoptotic protein activating-factor-1 (Apaf-1) plays an important role in mitochondrial pathway apoptosis, and Jujuboside A plays a therapeutic role by downregulating the expression of mitochondrial apoptotic proteins (Bax, Cyto c Apaf-1) (Zhong et al., 2022). *In vitro* and *in vivo* experiments, Aldose reductase (AR) is a NADPH-dependent oxidoreductase involved in the glucose metabolism polyol pathway. Recent studies (He et al., 2022) have confirmed that Ginsenoside Rb1 inhibits AR activity and its downstream factor, Recombinant Nicotinamide Adenine Dinucleotide Phosphate Oxidase 4 (NOX4), by binding to AR. Ginsenosaponin Rb1 directly or indirectly reduces NOX4 activity, inhibits ROS production, attenuates the release of Cyto c, and attenuates apoptosis, thus maintaining mitochondrial structural and functional integrity. *Astragalus* polysaccharide (APS) is one of the main active components of *Astragalus* and AMPK is a mitochondrial ATP energy receptor that can be activated when the AMP/ATP ratio increases. Subsequently, its downstream SIRT1/PGC-1α pathway was activated, and so far little research has been done on the role of APS in mitochondrial dysfunction in diabetic nephropathy. Using human proximal tubular epithelial cell line (HK-2) as a model, Xu (2020) found that APS upregulates ATP-containing and suppresses caspase-9 overexpression, confirming that APS mediates mitochondrial apoptosis through the AMPK/SIRT1/PGC-1α pathway.

## Protective effect of natural products in gouty nephropathy *via* regulating mitochondrial dysfunction

Gouty nephropathy (GN), also known as hyperuricemia nephropathy, is kidney damage caused by abnormal uric acid metabolism that causes crystals of uric acid to deposit in the distal tubules or pooled tubules. With the improvement of living standard, more and more patients with hyperuricemia and GN. Kidney damage occurs in almost all patients with gout, the report said. And hyper uric acid is not just a symptom associated with kidney disease, it can also be an independent risk factor for kidney damage. The pathogenesis of GN is not fully understood, but it is mainly due to a series of endothelial inflammatory reactions such as the accumulation of uric acid in renal tubules and renal stroma under hyper uric acid metabolism. The formation of mono-sodium uric acid crystals leads to the accumulation of large numbers of inflammatory cells and macrophages, which in turn leads to glomerular damage and renal fibrosis, and further development of interstitial nephritis, kidney failure. Meanwhile, high blood uric acid metabolism induces ROS, mitochondrial dysfunction, inflammatory response and renin-angiotensin system (RAS) activation which was also found to be involved in disease progression (Bidani and Griffin, 2004; Eirin and Lerman, 2013).

In recent years, the treatment of gouty nephropathy by natural products through mitochondrial pathway has attracted much attention, among which fucoidan can reduce serum uric acid, urea nitrogen and creatinine levels in rats. And in the pathological observation, can see the obvious improvement, manifests in the renal production of ROS decreased, the lysosomal production reduces, and alleviation of apoptosis (Deng et al., 2018). Quercetin has been found to partially improve mitochondrial damage in tubular epithelial cells, partially reduce damage to the renal microvascular system and relieve uric acid kidney damage by increasing the antioxidant capacity of the kidneys (Fen et al., 2021), this protective effect may be achieved by improving mitochondrial function through PI3K/AKT signaling, thereby reducing apoptosis and promoting cell proliferation. Phloretin relieves UA induced kidney injury by reducing mitochondrial OS and NLRP3 pathway mediated inflammatory response (Cui et al., 2020). The flavonoid-rich fraction from rhizomes of Smilax glabra Roxb (SGF) ameliorates mitochondrial damage in renal epithelial cells by significantly inhibiting IL-6, TNF-α, IL-1β, TGF-β1, and increasing the activities of antioxidant enzymes such as SOD, CAT and MDA to suppress inflammatory responses and OS (Wang S. et al., 2019). Thymoquinone was also found to reduce mitochondrial oxidative damage by increasing total ATP levels in kidney mitochondria and by activating the Nrf2/HO-1 signaling pathway (Dera et al., 2020). In short, natural products mostly improve kidney damage caused by uric acid by improving mitochondrial oxidative stress or inhibiting its downstream inflammatory factors. Combined pathogenesis, Improving UA metabolism is a potential therapeutic approach.

## Protective effect of natural products in hypertensive kidney disease *via* regulating mitochondrial dysfunction

Hypertensive nephropathy is the long-term increase in blood pressure that causes renal arterioles and small arteries, arterial stenosis and secondary ischemic renal parenchyma, glomerulosclerosis, tubule atrophy, and renal interstitial fibrosis. Clinical features include increased nocturnal urination, low specific weight urination, light to moderate albuminuria, and progressive decrease in glomerular filtration rate (GFR) leading to end-stage nephropathy. The study suggested that inflammation, OS, and fibrosis are the main risk factors. However, the molecular mechanisms are unclear (Bidani and Griffin, 2004). Renal damage caused by hypertension includes oxidative stress, renin-angiotensin-aldosterone system (RAAS), renal remodeling, and apoptosis, all of which impair mitochondrial integrity and function. NLRP3 protein located in mitochondria induces ROS *via* Smad (Bracey et al., 2014). Conversely, mitochondrial-derived ROS induces mitochondrial dysfunction, which further promotes NLRP3 inflammatory small-body activation (Che et al., 2014). Activation of NLRP3 inflammatory microspheres may induce proteinuria-induced tubule damage and promote TGF-β-induced phenotypic alterations in proximal tubule cells leading to hypertensive kidney damage (Zhuang et al., 2015; Gong et al., 2016). Therefore, the search for natural monomers that protect mitochondrial function and protect antioxidants has become a hot topic in recent years.

Chronic hypertension may lead to renal ischemia. Prem and Kurian (2022) found that Fisetin pretreatment protects kidney function by improving cysteine activity, reducing DNA fragmentation, retaining mitochondrial ETC activity, and increasing ATP production in kidney tissue during IR injury. The structural similarity between Fisetin and resveratrol predicts Fisetin’s ability to supply electrons to ETCs, thereby promoting proton drive (PMF) production, which may eventually lead to ATP production. Notably, by reversing PGC-1α gene expression, Fisetin reduced mitochondrial fission (Fis, Dynamin 1) and increased mitochondrial fusion (Mfn 1) and mitochondrial autophagy (Parkin and Optineurin) to protect mitochondrial mass in kidney tissue. CUR protects against kidney damage and inflammation caused by 5/6 nephrectomy (5/6 NX) in rats (Ghosh et al., 2009). Study found that this protective effect is due to curcumin’s direct and indirect antioxidant effects. These changes were accompanied by a decrease in renal content of several GSH and Nrf2-dependent enzymes and an increase in NADPH oxidase expression. Francisco Correa found that CUR has a direct antioxidant effect on organs and may be involved in downstream signaling of PKC activation (Correa et al., 2013). Magnesium lithospermatate B (MLB) derived from *Salvia miltiorrhiza Bunge*. The study suggested that MLB inhibited the oligomerization of Bax in mitochondria and releases Cyto c into the cytoplasm, showing anti-apoptotic effects in CRF rats, which may be associated with improved kidney damage. In the 5/6 (A/I) model, p53 phosphorylation and acetylation of lysine 379 may be involved in the mitochondrial pathway of apoptosis, and one of the mechanisms by which MLB inhibits the intrinsic pathway of apoptosis may be inhibition of p53 activation (Wang Y. et al., 2019). Mitochondrial dysfunction has been shown to lead to the production of reactive oxygen species (ROS), which mediates NLRP3 inflammatory small-body activation and leads to aldosterone-induced renal tubule cell injury (Ding et al., 2016). Ding et al. (2015) observed that Rotenone (ROT), an inhibitor of mitochondrial complex I, attenuated renal injury induced by aldosterone injection in rats by reducing SOD, NLRP3, IL-1β, and IL-18 activities.

Melatonin was isolated from the pineal gland of animals in 1958 and is found in almost all plants and plant products. Scholars believe melatonin is a powerful antioxidant and ROS scavenger that protects ETC and mtDNA from oxidative damage (Ramis et al., 2015). Melatonin was found to reduce blood pressure and kidney protection by reducing OS in the kidneys (Cheng et al., 2014).

Endothelial damage and OS associated with improved blood pressure are important in hypertensive kidney damage, further research is needed to determine whether natural products that control blood pressure can be potential targets (Stacchiotti et al., 2014).

## Protective effect of natural products in obesity-related nephropathy *via* regulating mitochondrial dysfunction

Obesity-associated global disease (ORG) The obesity epidemic has led to an increase in the incidence of obesity-related glomerular disease (ORG). Studies showed that the incidence of ORG increased gradually from 0.2% in 1986%–1990% to 2.7% in 2001–2015 (Kambham et al., 2001; Jia, 2015), And the number of patients is now growing. Mitochondrial dysfunction has been linked to obesity and obesity-related diseases and can trigger autophagy to protect kidney function (Mizushima, 2009). Similar to another metabolically relevant nephropathy, in Alessandra Stacchiotti’s study, ob/ob mice supplemented with melatonin altered mitochondrial shape and crest tissue in the proximal tubules, enhancing MFN-2 expression and thus modulating the progression of mitochondrial driven intrinsic apoptotic pathways. These changes may help reduce kidney failure.

A study found that autophagy ablation enhances HFD-induced mitochondrial dysfunction and activation of small inflammatory bodies, suggesting that HFD-impaired autophagy flux contributes to renal lip toxicity remission (Yamamoto et al., 2017). The fatty tissue accumulated in obesity stimulates the production of pro-inflammatory cytokines and bioactive substances called “adipokines” that increase ROS production. Inflammation, OS, and mitochondrial dysfunction contribute to the pathogenesis of metabolic disorders, including insulin resistance and T2D (Sivitz and Yorek, 2010; de Mello et al., 2018). Studies have shown that hypertriglyceridemia promotes LDL deposition and reduces HDL levels, increasing the risk of progression of chronic kidney disease (Lanktree et al., 2018). With these studies in mind, targeting mitochondria may lead to new strategies for ORG treatment. Rhein, for example, corrects hypercholesterolemia by downregulating the FAT/CD36 table, reduces triglyceride levels to control lipid metabolic disorders, and mitochondrial morphology and function are restored under electron microscopy (Huang et al., 2004). As a SIRT1 agonist, RESV significantly upregulated SIRT1 protein expression and upregulated GPx4 and SOD2 protein expression in renal tissues. Regulating lipid metabolism disorders to remove excess ROS and improve ROS and ERS associated with high fat. Improve the damage of mitochondrial structure and function of renal epithelial cells, and play a protective role in kidney (Xu et al., 2019).

Obesity-related nephropathy has many similar mechanisms to diabetic nephropathy and the efficacy of many natural products such as resveratrol and melatonin deserves further study.

## Conclusion and future perspectives

**FIGURE 1:**
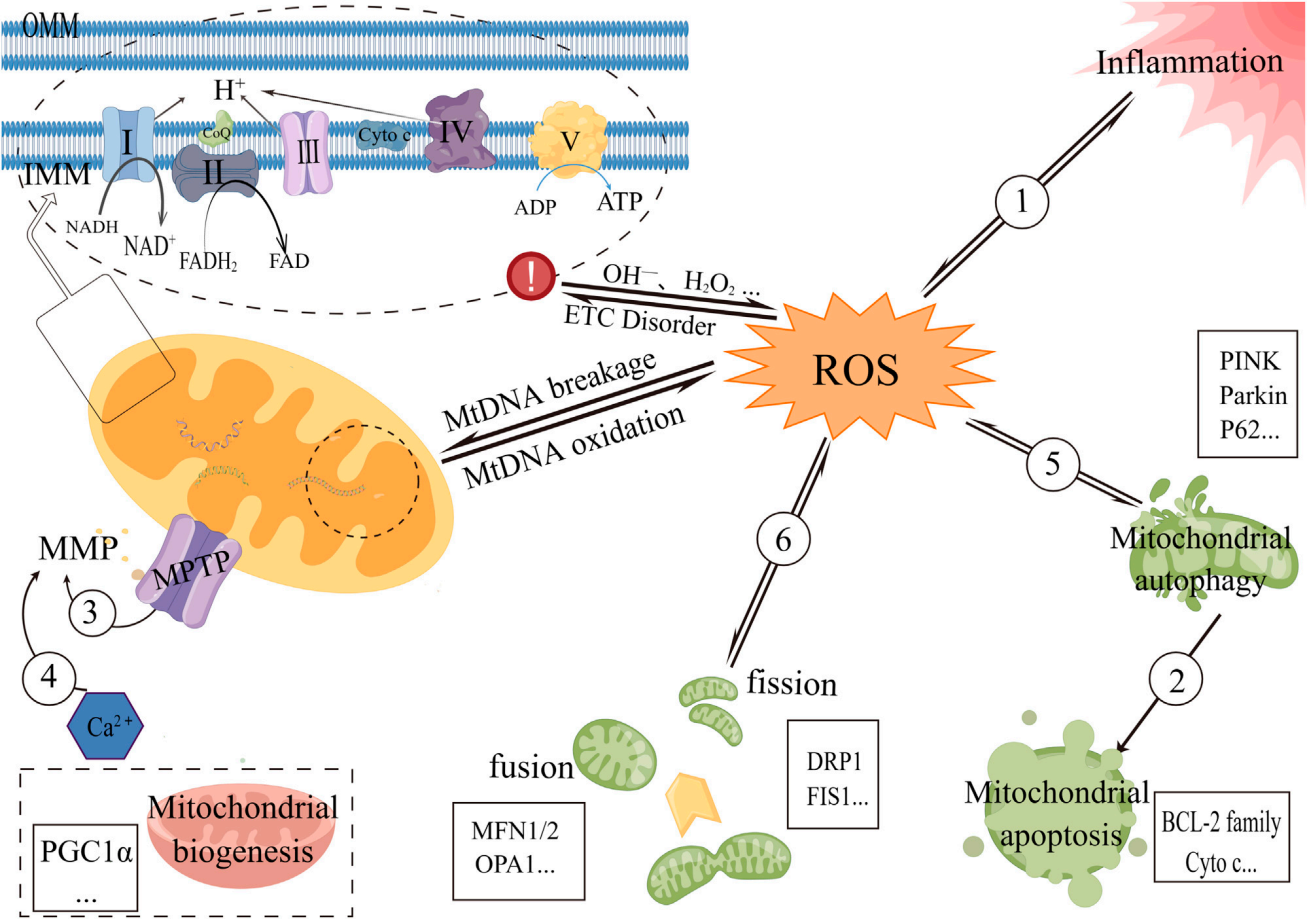
Diagram of some mechanisms of mitochondrial dysfunction in metabolic-associated kidney diseases 1) Oxidized mtDNA fragments can enter the cytoplasm *via* MPTP and then bind to NLRP3 and cyclic guanosine monophosphate-adenosine monophosphate synthase (cGAS), which can also activate the NLRP3 inflammasome. ROS can also induce exacerbation of inflammation. 2) Autophagy of the mitochondrial pathway disrupts the mitochondrial bilayer structure and activates apoptosis-related proteins, causing apoptosis in the mitochondrial pathway. 3) Increased MPTP opening results in the release of negatively charged proteins into the mitochondrial matrix, which affects ion concentrations on both sides of the mitochondrial inner membrane and, ultimately, causes MMP levels to drop. 4) Ca2^+^ triggers a common mitochondrial membrane permeability transition (MPT), resulting in abnormal MPTP opening, mitochondrial swelling, and decreased membrane potential. 5) Mitochondrial autophagy maintains the integrity of mitochondrial function and reduces ROS production, and in turn, excess ROS can selectively degrade oxidatively damaged mitochondria by preferentially activating mitochondrial autophagy, thereby reducing its damage to cells. 6) Excessive ROS-induced oxidative stress decreases mitochondrial fusion and increases fission. Mitochondrial fission leads to changes in the mitochondrial membrane that fragment the mitochondria and also lead to an increase in ROS.

**FIGURE 2:**
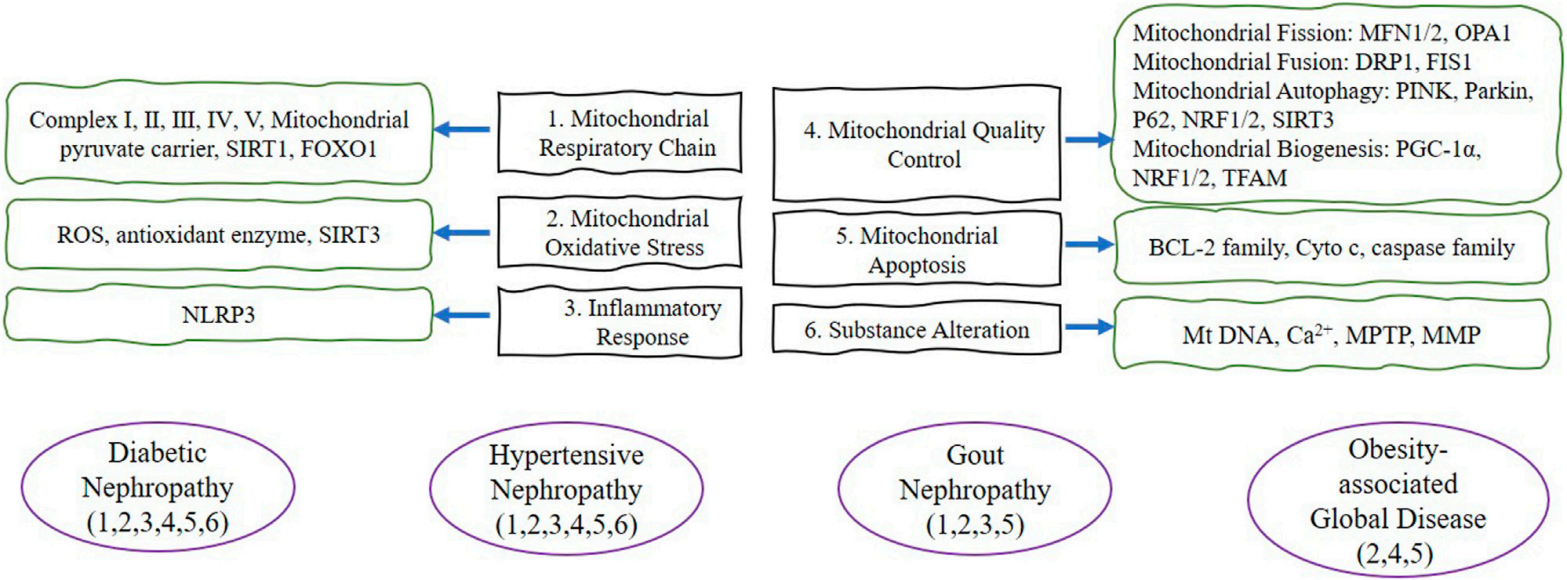
The purple lines represent the four disease types in this review. The green lines briefly depict the target proteins corresponding to the mitochondrial dysfunction in this review. The black frame describes the six mitochondrial dysfunctions summarized in this review. Abbreviation: SIRT1 (Silent information regulator 1), FOXO1 (forkhead transcription factor-O1), ROS (reactive oxygen species), SIRT3 (Silent information regulator 3), NLRP3 (NOD-like receptor protein 3), MFN1/2 (Mitofusin 1/2), OPA1 (Optic atrophy 1), DRP1 (dynamin-related protein 1), FIS1 (Fission 1), PINK (PTEN induced putative kinase 1), P62 (SQSTM1, sequestosome 1), NRF1/2 (nuclear response factor 1/2), PGC-1α (Peroxisome proliferator-activated receptor-gamma coactivator-1alpha), TFAM (transcription factor a mitochondrial), BCL-2 (anti-apoptotic protein B-cell lymphoma-2), Cyto c (cytochrome c), MtDNA (mitochondrial DNA), MPTP (Mitochondrial permeability transition pore), MMP (mitochondrial membrane potential).

With the increasingly important pathogenic role of OS in metabolic-associated kidney diseases, mitochondria, which are strongly associated with OS, are gradually gaining attention. Current studies have shown that the mechanisms triggering mitochondrial dysfunction include abnormal respiratory chain and energy metabolism, OS and inflammatory response, mitochondrial quality control, imbalance in mitochondrial dynamics, changes in mitochondrial material, reduced mitochondrial membrane potential, excessive autophagy and apoptosis of mitochondrial pathways (Figure 1 and Figure 2). Further study of the mechanisms of these may help in the development of drugs for mitochondrial dysfunction in metabolic-associated kidney diseases. However, this process is long. This paper reviews natural products that have been studied in recent years to improve metabolic-associated kidney diseases and are expected to be potential drugs for the treatment of metabolic-associated kidney diseases, mainly from the perspective of reducing mitochondrial dysfunction (Table 1).

**TABLE 1:** Natural product improve mitochondrial dysfunction in metabolic-associated kidney diseases.

Natural products	*In Vivo/In Vitro*	Model	Dose	Negative/positive control	Targets and pathways	References
Melatonin	*Both*	STZ-induced mice; HBZY-1 cells	20 mg/kg/d; high glucose	—	Cyto c/Bcl-2/Caspase	Satari et al. (2021)
			medium (25 mmol/L + 5 µM Melatonin		-3/9	
	*In Vivo*	L-NAME/SHR rat	SHRs received L-NAME (80 mg/L) and 0.01% melatonin in drinking water	NC: SHRs received L-NAME (80 mg/L) in drinking water	DDAH/ADMA	Cheng et al. (2014)
	*In Vitro*	Primary human kidney mesangial (HM) cells	0.5–100 µM	PC: rotenone (5 µM); antimycin A (5 µM); myxothiazol (5 µM)	Complex I, III Protein	Zhang et al. (2011)
	*In Vivo*	Male Zücker diabetic fatty (ZDF) rats and lean littermates (ZL)	10 mg/kg BW/day	—	MFN2, v, DRP1	Agil et al. (2020)
	*In Vitro*	Mouse podocytes	different concentrations of melatonin	—	MMP, ROS, Bax/Bcl-2, Caspase-3	Ji and Xu, (2016)
RESV	*Both*	STZ-induced mice, Conditional immortalized mouse podocytes	30 mg/kg/day; 10 μmol/L RESV	NC:0.5% carboxymethyl cellulose; NC:10 μmol/L EX527	Complex I, III Protein, ROS, MMP, Mn-SOD, Cyto C, SIRT1/PGC-1α	Zhang B. et al. (2019)
	*In Vivo*	db/db mice; Murine proximal tubular cells (mProx)	RESV was mixed (0.3%) with chow	—	ROS, Mn-SOD, mtDNA (D-17, 8-OHdG)	Kitada et al. (2011)
	*In Vivo*	C57BL/6J mice	5 mg/(kg·d)	—	SIRT1, SOD2, GPx4	Xu et al. (2019)
Notoginsenoside R1	*In Vivo*	Db/db mice	30 mg/kg/day	PC: metformin (200 mg/kg/day)	ROS, MMP, Bax/Bcl-2, Caspase-3, Caspase-9	Zhang T. et al. (2019)
Luteolin	*In Vitro*	mouse podocyte cell-5 (MPC-5)	various concentrations of luteolin	—	ROS, NLRP3, MMP, Bcl-2	Yu et al. (2019)
Syringic Acid	*In Vivo*	STZ-induced rats	25, 50, 100 mg/kg	—	PGC-1α, NRF-2 mRNA, NRF-1, mtDNA/nDNA ratio	Rashedinia et al. (2020)
	*In Vivo*	STZ-induced rats	25, 50, 100 mg/kg	—	CAT, SOD	Rashedinia et al. (2021)
Honokiol	*In Vivo*	Ob/ob mice	honokiol (10 mg/kg in DMSO)	NC: vehicle (DMSO in saline solution)	SIRT3, SOD2, restored PGC-1α	Locatelli et al. (2020)
Grape seed proanthocyanidins extract	*In Vivo*	STZ-induced SD rats	Distilled water + GSPE (125mg/kgbw, 250 mg/kgbw. 500mg/kgbw)	NC:Distilled water	Mtdna, AMPK-SIRT1-PGC-1α	Bao et al. (2014)
Cyanidin-3-O-β- glucoside chloride	*Both*	db/db mice; HK-2 cells	30 mg/kg body weight/day; 50 μM	NC: normal saline (once per day); NC: mannitol (24.4 mM)	ROS, MMP, Bax/Bcl-2, Caspase-3	Wei et al. (2018)
Curcumin	*In Vivo*	DN Mice	200 mg/kg, 400 mg/kg	PC: valsartan (20 mg/kg)	Complex I, III Protein, p-mTOR, Beclin1, ROS, Mn-SOD	Han et al. (2021)
	*In Vivo*	STZ-induced type 1 diabetes mellitus (DM) in rats	100 mg/kg	—	ROS, Mn-SOD, PKCβ/p66Shc/NADPH, Caspase-3, Cyto C	Altamimi et al. (2021)
	*Both*	db/db mice; HK-2 cells	200 mg/kg/d; 10 μm	NC:0.5% CMC; NC:5mM, 35 mM glucose model group	ROS, NLRP3	Lu et al. (2018)
	*In Vivo*	Sprague-Dawley rats was induced by 5/6 NX	75 mg/kg	PC:enalapril (10 mg/kg), NC: 0.5% CMC	CAT, GR, GPx, GST, SOD, Nrf2	Ghosh et al. (2009)
	*In Vivo*	Wistar rats was induced by 5/6 NX	120 mg/kg/day	NC: 0.5% CMC	SOD, CAT, GPx, GSH、IL-10, IFN-γ, MDA, IL-8, TNF-α, IL-6, MPO	Correa et al. (2013)
Astaxanthin	*In Vitro*	Exposure to high glucose levels NHMCs (human mesangial cell)	10^−7^–10^–4^ M Astaxanthin in 0.05% DMSO in PBS with 5% FBS	NC: 0.05% DMSO in PBS with 5% FBS	Peroxidase, Nrf2-ARE, MCP-1, TGFβ1	Manabe et al. (2008)
Puerarin	*In Vivo*	STZ-induced diabetic mice	20, 40, 80 mg/kg/day	PC: metformin (200 mg/kg)	SIRT1, FOX-O1α, PGC1α, NF-κB, IL-6, TNF-α	Xu et al. (2016)
Myriocin	*In Vivo*	Otsuka Long Evans Tokushima Fatty (OLETF) rats and high-fat diet (HFD)-fed mice	0.3 mg/kg/day	—	ROS	Woo et al. (2020)
Artemether	*In Vivo*	db/db mice	0.67 g/kg/d	—	MPC1, PGC-1α	Han et al. (2019)
	*In Vivo*	STZ-induced T1D mice	0.67 g/kg/d	—	PDK1, SOD2, catalase	Ni et al. (2017)
Icariin	*Both*	STZ-induced rats and HG-treated MPC-5 cells	20 mg/kg, 40 mg/kg, 80 mg/kg; 1 μM, 3 μM, 10 μM	PC: irbesartan (13.5 mg/kg; 1 μM)	Nrf2, NLRP3, Sesn2, Keap1	Ding et al. (2022)
Astragaloside II	*In Vivo*	STZ-induced rats	3.2, 6.4 mg/kg/d	Losartan (10 mg/kg/d)	MFN2, FIS1, LC3, caspase-3, P62	Su et al. (2021)
Berberine	*Both*	Conditionally immortalized mouse podocyte; db/db mice	0.4 μmol/L; 300 mg/kg/d	NC: 10 μmol/L Mdivi1	PGC1α, TFAM, NRF1, NRF2, Drp1	Qin et al. (2019)
Polydatin	*In Vivo*	KKAy mice and MPC5	—	—	DRP1, ROS	(Ni et al., 2017)
Astragaloside IV	*In Vivo*	db/db mice	standard feed at a dose of 1 g/kg Astragaloside IV	—	Drp-1, Fis-1, MFF, PINK1, Parkin, LC-3II	Liu et al. (2017)
	*In Vivo*	db/db mice	2, 6, 18 mg/kg/d	PC: rosiglitazone (2 mg/kg/d), NC:vehicle	PPAR-γ/SECRCA2/Ca^2+^, MPTP	Guo et al. (2016)
Purslane polysaccharide	*In Vivo*	STZ-induced rats	100 mg/kg, 200 mg/kg, 400 mg/kg	—	Complex I, III Protein, Mn-SOD	Wang and Ye, (2018)
Quercetin	*In Vitro*	GNR8 rats, NRK-52E cells	diabetic rats treated with quercetin (25, 50 and 100 mg/kg)	NC: allopurinol (10 mg/kg)	PI3K, AKT, mTOR	Fen et al. (2021)
				PC:quercetin (25, 50 and 100 mg/kg)		
Phloretin	*Both*	Hyperuricemia (HUA)-induced renal injury in mice, human renal tubular cell lines (HK-2)/HUVECs	50 mg/kg	NC:0.5% CMC-Na;-	ROS, NLRP3, GLUT9	Cui et al. (2020)
			in 0.5% CMCeNa daily;-			
The flavonoid-rich fraction from rhizomes of Smilax glabra Roxb	*In Vivo*	Sprague Dawley (SD) rats were induced by high purine diet (yeast pellets + adenine)	Yeast pellets + adenine 50 mg/kg + SGF 100 mg/kg/SGF 300 mg/kg/SGF 500 mg/kg	NC: yeast pellets + adenine 50 mg/kg + allopurinol 40 mg/kg	CAT, MDA, ROS	Wang M. et al. (2019)
Jujuboside A	*In Vivo*	DN rats	JuA (20 mg/kg)	NC: Metformin (Met, 300 mg/kg)	SOD, CAT, GPx	Zhong et al. (2022)
Taurine	*Both*	STZ-induced DKD model and MPC-5 cells	taurine (2%, in drinking water); -	—	ROS, CSE, TRPC6	Zhang et al. (2020)
	*In Vivo*	Alloxan (single i.p. Dose of 120 mg/kg body weight) induced DN rats	Taurine (1% w/v in drinking water)	—	Bax/Bad, Bcl-2/Bcl-xL, cytoc, caspase-9, caspase-3	Das and Sil, (2012)
Rhein	*In Vivo*	Db/db mice	Rhein (120mg/(kg·d))	NC: Rosiglitazone [4mg/(kg·d)]	PPARγ, resistin FAT/CD36	Huang et al. (2004)
Thymoquinone	*In Vivo*	OA 750 mg/kg BW for 12 weeks was used to induce uricemia in SD rats	Thymoquin (10, 20 mg/kg BW)	—	ROS, Akt, Nrf2, HO-1, caspase-3	Dera et al. (2020)
Fisetin	*In Vivo*	Renal ischemic reperfusion (IR) injury in Male Wistar rats	Fisetin (20 mg/kg b.wt, ip)	—	GSH/GSSG, SOD, CAT, GPx, G, PGC-1α, Mff, Fis 1, Dnm 1, MPTP	Prem and Kurian, (2022)
Magnesium lithospermatate B	*In Vivo*	5/6NX in SD rats	—	—	Caspase-3, Bax, cytochrome c, p53	Wang S. et al. (2019)
Rotenone	*In Vivo*	Underwent right uninephrectomy SD rats was infused aldosterone	Rotenone 600 ppm	NC: vehicle	SOD, MDA, NLRP3, IL-1β, and IL-18	Ding et al. (2015)
Salidroside	*In Vivo*	STZ mice	50, 100 mg/kg/day	PC: Metformin (0.6 mg/mL)	SIRT1/PGC-1α	Xue et al. (2019)

Natural products have multi-component, multi-target and multi-level characteristics and can be used to treat metabolic-associated kidney diseases in different stages. Compared to most chemical agonists, natural products may not have the thermodynamic advantage to achieve favorable binding properties. Therefore, the scientific community is increasingly interested in natural products due to their pharmacological and economic advantages. Therefore, natural products are an excellent complementary therapy for the treatment of mitochondrial dysfunction in metabolic-associated kidney diseases.

However, there are still disadvantages to using natural products. At present, the clinical efficacy data of natural products on metabolic-associated kidney diseases are mainly concentrated in Asia, and there is a lack of data on the efficacy results in other regions. It is difficult to estimate the difference between different regions based on animal models. Consequently, it is impossible to fully prove that natural products are effective for metabolic-associated kidney diseases patients in different regions. In this respect, it is important to note that the toxicity measures of natural products to humans are not well simulated in animal studies.

Collectively, detailed information on the pharmacology, potential toxicity and side effects of natural products needs to be provided prior to their use. In addition to preclinical studies, clinical studies are necessary for the treatment of metabolic-associated kidney diseases with natural products. As mentioned above, natural products usually have two major drawbacks - limitations of clinical application and potential toxicity-both of which require researchers to find further solutions. Not only do natural products have relatively easy access and low toxicity, but more importantly they may have significant efficacy in the treatment of metabolic-associated kidney diseases.
